# Site-Directed Mutagenesis Improves the Transduction Efficiency of Capsid Library-Derived Recombinant AAV Vectors

**DOI:** 10.1016/j.omtm.2020.03.007

**Published:** 2020-03-13

**Authors:** Gai Ran, Xiao Chen, Yilin Xie, Qingyun Zheng, Jinyan Xie, Chenghui Yu, Nikea Pittman, Sixian Qi, Fa-Xing Yu, Mavis Agbandje-McKenna, Arun Srivastava, Chen Ling

**Affiliations:** 1 State Key Laboratory of Genetic Engineering, School of Life Sciences, Zhongshan Hospital, Fudan University, Shanghai 200438, China; 2 Department of Biochemistry and Molecular Biology, University of Florida College of Medicine, Gainesville, FL 32611, USA; 3 Powell Gene Therapy Center, University of Florida College of Medicine, Gainesville, FL 32611, USA; 4 Genetics Institute, University of Florida College of Medicine, Gainesville, FL 32611, USA; 5 Division of Cellular and Molecular Therapy, Department of Pediatrics, University of Florida College of Medicine, Gainesville, FL 32611, USA; 6 Department of Molecular Genetics and Microbiology; 7 Shands Cancer Center, University of Florida College of Medicine, Gainesville, FL 32611, USA; 8 Institute of Pediatrics, Children’s Hospital of Fudan University, Shanghai 201102, China; 9 Shanghai Key Laboratory of Medical Epigenetics, Institutes of Biomedical Sciences, Fudan University, Shanghai 200032, China

**Keywords:** gene therapy, rAAV vector, library selection, site-directed mutagenesis, transduction efficiency, vector distribution, hepatocellular carcinoma, cancer targeting

## Abstract

Recombinant adeno-associated virus (rAAV) vectors selected from capsid libraries present enormous advantages in high selectivity of tissue tropism and their potential use in human gene therapy applications. For example, rAAV-LK03, was used in a gene therapy trial for hemophilia A (ClinicalTrials.gov: NCT03003533). However, high doses in patients resulted in severe adverse events and subsequent loss of factor VIII (FVIII) expression. Thus, additional strategies are needed to enhance the transduction efficiency of capsid library-derived rAAV vectors such that improved clinical efficacy can be achieved at low vector doses. In this study, we characterized two commonly used library-derived rAAV vectors, rAAV-DJ and rAAV-LK03. It was concluded that rAAV-DJ shared similar transport pathways (e.g., cell surface binding, endocytosis-dependent internalization, and cytoplasmic trafficking) with rAAV serotype 2, while rAAV-LK03 and rAAV serotype 3 shared similar transport pathways. We then performed site-directed mutagenesis of surface-exposed tyrosine (Y), serine (S), aspartic acid (D), and tryptophan (W) residues on rAAV-DJ and rAAV-LK03 capsids. Our results demonstrated that rAAV-DJ-S269T and rAAV-LK03-Y705+731F variants had significantly enhanced transduction efficiency compared to wild-type counterparts. Our studies suggest that the strategy of site-directed mutagenesis should be applicable to other non-natural AAV variants for their optimal use in human gene therapy.

## Teaser

Recombinant adeno-associated virus (rAAV) vectors derived from the capsid library have shown great promise to treat human diseases. Still, their transduction efficiency is the major challenge in therapeutic effects. Ling and colleagues demonstrated that multiple site-directed mutations on capsid library-derived rAAV vectors ameliorate transduction efficiency both *in vitro* and *in vivo*.

## Introduction

With the features of non-pathogenic nature, wide tissue tropism, and long-term transgene expression, recombinant adeno-associated virus (rAAV) vectors have gained significant attention. They have shown great promise in the treatment for several monogenic human diseases, including neurodegenerative and metabolic disorders.^1^ Despite these remarkable achievements, the most significant obstacle is the host humoral and cellular immune response.^2^ It is thought that if the vector dose can be kept to a minimum, without compromising its efficacy, this obstacle can be circumvented. Efforts are currently ongoing in a number of laboratories, including our own, to achieve high-efficiency transduction at reduced vector doses, which include the following: (1) use of rAAV vectors with a natural tropism for the target cell/tissue/organ;^3^ (2) rational design of viral capsid proteins with site-directed mutagenesis^4^ and peptide insertion;^5^ and (3) selection from viral libraries that contain millions of chimeric capsids.^6^^,^^7^

Among the rAAV variants, rAAV-DJ and rAAV-LK03 are two chimeric vectors created from capsid libraries using DNA shuffling technology. rAAV-DJ was selected in the presence of pooled human antisera, followed by validation of high transduction efficiency with the ability to evade immune neutralization compared to other serotypes.^8^ It is a chimera of serotypes 2, 8, and 9. It is distinguished from its closest natural relative AAV2 by 60 amino acids. Since its discovery, rAAV-DJ has been broadly used in gene delivery, such as to deliver a knockout construct to fetal pig fibroblasts for the production of Fah-null heterozygote pigs,^9^ and to mediate gene targeting in keratinocytes by homologous recombination.^10^ rAAV-LK03 was selected using liver humanized mice.^11^ The chimeric capsid is composed of nucleotide sequences from seven different parental AAV genomes (AAV1, 2, 3B, 4, 6, 8, and 9). It represents 97.7% homology of the capsid gene sequence and 98.9% of the capsid amino acid sequence with the rAAV3B capsid. In 2016, Spark Therapeutics sponsored a human clinical trial (ClinicalTrials.gov: NCT03003533) using the rAAV-LK03 vector to treat hemophilia A patients.^12^ Although clinical efficacy was achieved at a relative low dose, at least two patients experienced severe adverse events at a higher vector dose.

A more detailed understanding of transduction mechanisms of chimeric rAAV vectors is warranted to achieve improved efficiency at lower doses. Various steps in the life cycle of AAV vectors include attachment to cell surface receptors, endocytosis, intracellular trafficking, nuclear translocation, uncoating, and conversion of single-stranded DNA (ssDNA) genome to double-stranded DNA (dsDNA) prior to transgene expression.^13^ The use of specific pharmacological inhibitors of each step has helped reveal the intricacies of the vector-mediated transduction mechanisms (Table 1). It is of note that most experiments involving specific pharmacological inhibitors have been performed using rAAV serotype 2, a prototype in the field. Since it is unclear whether the transduction efficiency of these library-derived rAAV vectors could be further improved by strategies of rational design, we carried out systematic studies to gain a better understanding of the transport pathways of rAAV-DJ and rAAV-LK03 from attachment to cellular receptors and intracellular trafficking. Based on our observation and previous studies, we further present experimental evidence herein that site-directed mutagenesis of specific amino acids on the rAAV-DJ and LK03 capsids leads to further enhancement in the transduction efficiency. Thus, it is possible to achieve improved efficiency at lower vector doses in order to circumvent the host cellular immune response.

**Table 1:** Specific Pharmacological Inhibitors Have Helped Reveal the Intricacies of the Vector-Mediated Transduction Mechanisms

Name	Functions	References
Heparin	closely related in structure to membrane heparan sulfate	^34^^,^^35^
Jasplakinolide	prevents actin depolymerization	^36^
5-(*N*-ethyl-*N*-isopropyl) amiloride (EIPA)	inhibits CLIC/GEEC endocytosis as an inhibitor of Na^+^/H^+^ exchange of cytomembrane	^19^^,^^36^
Bafilomycin A1	a lysosome acidification inhibitor	^15^
Brefeldin A	inhibits protein transport from the Golgi apparatus to the endoplasmic reticulum	^37^^,^^38^
MG132	prevents cellular proteasome-mediated degradation	^15^

## Results

### Infectious Pathway of rAAV-DJ Vectors in HEK293 Cells

**Figure 1:**
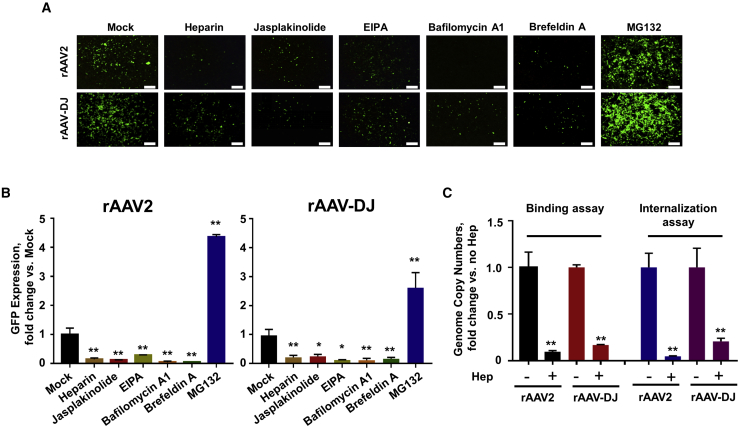
Transduction Efficiency of rAAV2-CMV-*egfp* and rAAV-DJ-CMV-*egfp* Vectors in HEK293 Cells, Treated by Various Specific Pharmacological Inhibitors (A) HEK293 cells were infected with GFP-expressing rAAV2 or rAAV-DJ in the presence of specific inhibitors (100 μg/mL heparin, 2 μM jasplakinolide, 10 μM EIPA, 200 nM bafilomycin A1, 10 μg/mL brefeldin A, and 20 μM MG132). GFP expression was measured at 24 h post-infection by fluorescence microscope. Scale bars represent 300 μm. (B) Quantitation of transduction efficiency of rAAV2 and rAAV-DJ with different inhibitors. Data were normalized by rAAV2- and rAAV-DJ-infected and mock-treated groups, respectively. (C) Binding and internalization assays were performed using vector-transduced HEK293 cells with or without heparin-treatment. All values shown are means ± standard deviations. ∗p < 0.05, ∗∗p < 0.01 versus mock.

rAAV-DJ vector efficiently transduces a broad spectrum of cells *in vitro*, with the highest potency in HEK293 cells.^8^ Thus, we used HEK293 cells as a model system to investigate various steps in the life cycle of rAAV-DJ vectors. Consistent with other reports,^14^ rAAV-DJ-CMV (cytomegalovirus)-*egfp* vectors transduced HEK293 cells better than did rAAV2-CMV-*egfp* vectors (Figure 1A). We observed inhibition of the rAAV2 and rAAV-DJ transduction with 100 μg/mL heparin treatment, suggesting that the heparan sulfate proteoglycan (HSPG) is essential for both vectors (Figure 1B). More importantly, binding and internalization assays also showed that heparin strongly blocked rAAV2 and rAAV-DJ binding to the cell surface and affected the number of viral particles entering the cells (Figure 1C). To analyze the endocytosis pathways utilized by each vector, both 2 μM jasplakinolide, which is known to prevent actin depolymerization, and 10 μM 5-(*N*-ethyl-*N*-isopropyl) amiloride (EIPA), which has been shown to inhibit CLIC (clathrin-independent carrier)/GEEC (glycosylphosphatidylinositol-anchored protein-enriched early endosomal compartment)-mediated endocytosis, were used and proved to inhibit rAAV2- and rAAV-DJ vector-mediated transduction. We also observed that 200 nM bafilomycin A1, a specific inhibitor of endosome acidification, eliminated rAAV2 transduction, suggesting that intracellular trafficking was involved.^15^ At the same or higher concentration of bafilomycin A1, rAAV-DJ vector still retained infectivity, although significantly reduced. This indicated that rAAV-DJ vector is more tolerant of changes in endosomal pH. Treatment with 10 μg/mL brefeldin A, a Golgi assembly inhibitor, dramatically inhibited rAAV-DJ vector transduction, an observation similar to the rAAV2 vectors. Finally, a specific proteasome inhibitor MG132 dramatically enhanced the rAAV2- and rAAV-DJ-mediated transgene expression in HEK293 cells, suggesting that proteasome-mediated degradation negatively impacts the transduction efficiency of both vectors (Figures 1A and 1B).

### Site-Directed Mutagenesis of rAAV-DJ Capsid Improves Transgene Expression *In Vitro*

**Figure 2:**
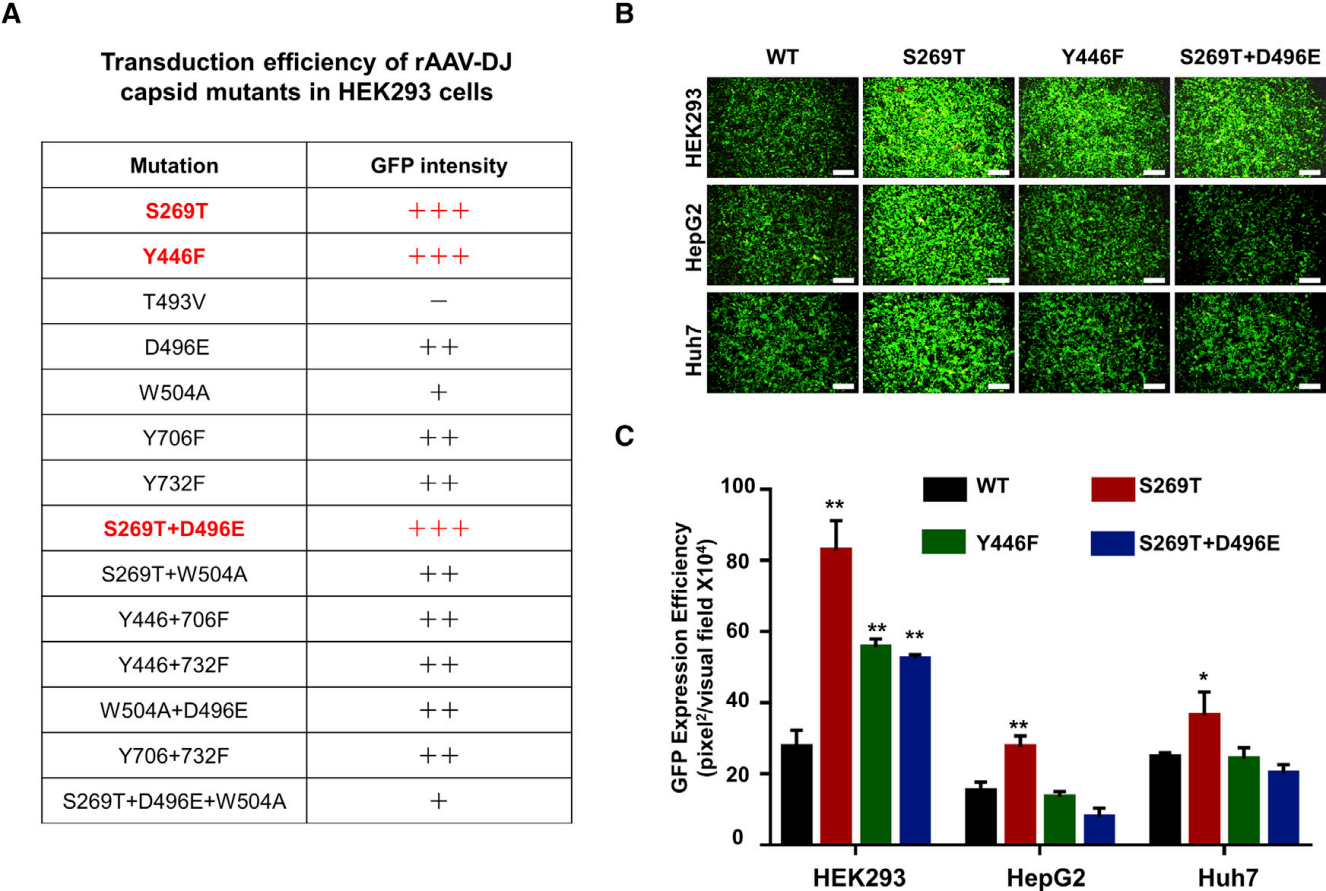
Transduction Efficiency of Site-Directed rAAV-DJ Capsid Mutants in Human Cell Lines (A) +++, significant increase; ++, no differences; +, significant reduction; −, complete loss of transgene expression; versus WT. (B) HEK293, Huh7, and HepG2 cell lines were transduced with the WT, S269T, Y446F, or S269T+D496E mutant rAAV-DJ-CMV-*egfp* vectors at an MOI of 5,000 vg/cell. Transgene expression was analyzed by fluorescence microscopy 72 h post-transduction. (C) Quantitation of transduction efficiency of rAAV-DJ mutants in cell lines. All values shown are means ± standard deviations. ∗p < 0.05, ∗∗p < 0.01 versus WT.

Since the rAAV2 vector shared similar transport pathways with the rAAV-DJ vector, we chose to mutate the same sites on the rAAV-DJ capsid (Y446, T493V, Y706, Y732, S269, D496, W504), as those were published on the rAAV2 capsid. The variant capsids, along with their wild-type (WT) counterpart, were used to package a self-complementary (sc) viral genome including an *egfp* gene under the control of a CMV promoter. No significant differences in the packaging efficiency were observed, as determined by quantitative polymerase chain reaction (qPCR) assays of purified viral stocks (data not shown). The transduction efficiency of all mutant vectors was tested in the HEK293 cells. As shown in Figure 2A, GFP intensity results documented that the rAAV-DJ-S269T mutant was the most efficient, followed by S269T+D496E and Y446F mutants. The T493V mutant failed to transduce cells, and the single mutant of W504 or its combination with other mutants also dramatically reduced the transgene expression. Furthermore, the transduction efficiencies of the top three capsid mutants were compared in various cell lines side by side. The results showed that the rAAV-DJ-S269T mutant increased the transduction efficiency by 3-, 1.8-, and 1.5-fold in the HEK293, HepG2, and Huh7 cells, respectively, compared with the WT AAV-DJ vectors. However, the transduction efficiency of the other two mutants, Y446F and S269T+D496E, which were efficient in transducing HEK293 cells, was not significantly improved in human hepatocellular carcinoma cell lines (Figures 2B and 2C). These results nonetheless corroborate that it is possible to increase the transduction efficiency of rAAV-DJ vectors following capsid modifications.

### *In Vivo* Characterization of the rAAV-DJ-S269T Vectors

**Figure 3:**
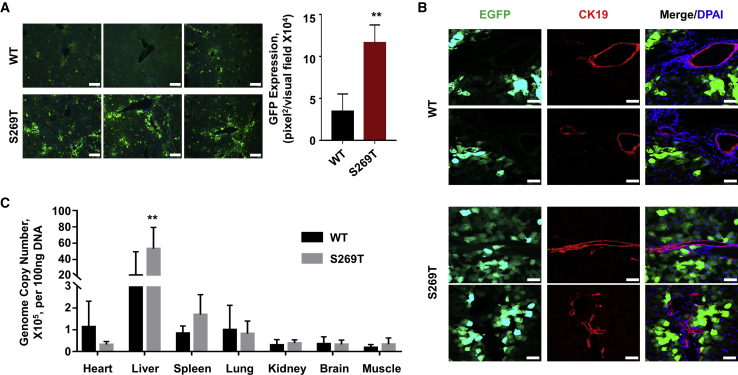
Transduction Efficiency of rAAV-DJ-WT and rAAV-DJ-S269T Vectors *In Vivo* Mice were tail-vein injected with the indicated vectors containing a CMV-*egfp* gene at 1 × 10^11^ vg/mouse (n = 3). Major tissues were obtained at 4 weeks post-injection. (A) rAAV-DJ-S269T mediated higher *egfp* expression, detected by fluorescence microscopy of liver sections. Quantitative data are presented. Scale bars represent 200 μm. (B) Immunofluorescence staining of liver sections at ×400 original magnification. The merged images showed that both vectors preferentially transduce hepatocytes, but not CK19^+^ cholangiocytes. Green indicates EGFP; red indicates CK19; blue indicates DAPI staining for nucleus. Scale bars represent 50 μm. (C) Tissue DNA was isolated and qPCR was performed to measure the AAV genome copies per 100 ng of DNA. All values shown are means ± standard deviations. ∗∗p < 0.01 versus WT.

The WT rAAV-DJ and mutant rAAV-DJ-S269T vectors containing an *egfp* gene were tail vein injected into the C57BL/6 mice at a dose of 10^11^ vector genomes (vg)/mouse. At 4 weeks post-injection, liver tissues were harvested and sections of each of the lobes were examined for EGFP expression. We observed a significant increase in the transgene expression in the mice liver that were administered with rAAVDJ-S269T vectors (Figure 3A). Cell type-specific tropism was also determined by immunofluorescence staining. These results, shown in Figure 3B, clearly indicated that neither vector efficiently transduced cholangiocytes, another important cell population in the liver.^16^ The AAV vector genome biodistribution profiles in all study animals were also assessed by qPCR. The vector genomes of both mutant rAAV-DJ-S269T vectors and their parental WT rAAV-DJ vectors were predominantly enriched in the liver (Figure 3C). Most importantly, mutant vectors resulted in significantly increased genome copy number compared to the WT vectors in the liver (Figure 3C).

### Infectious Pathway of rAAV-LK03 Vectors in Huh7 Cells

**Figure 4:**
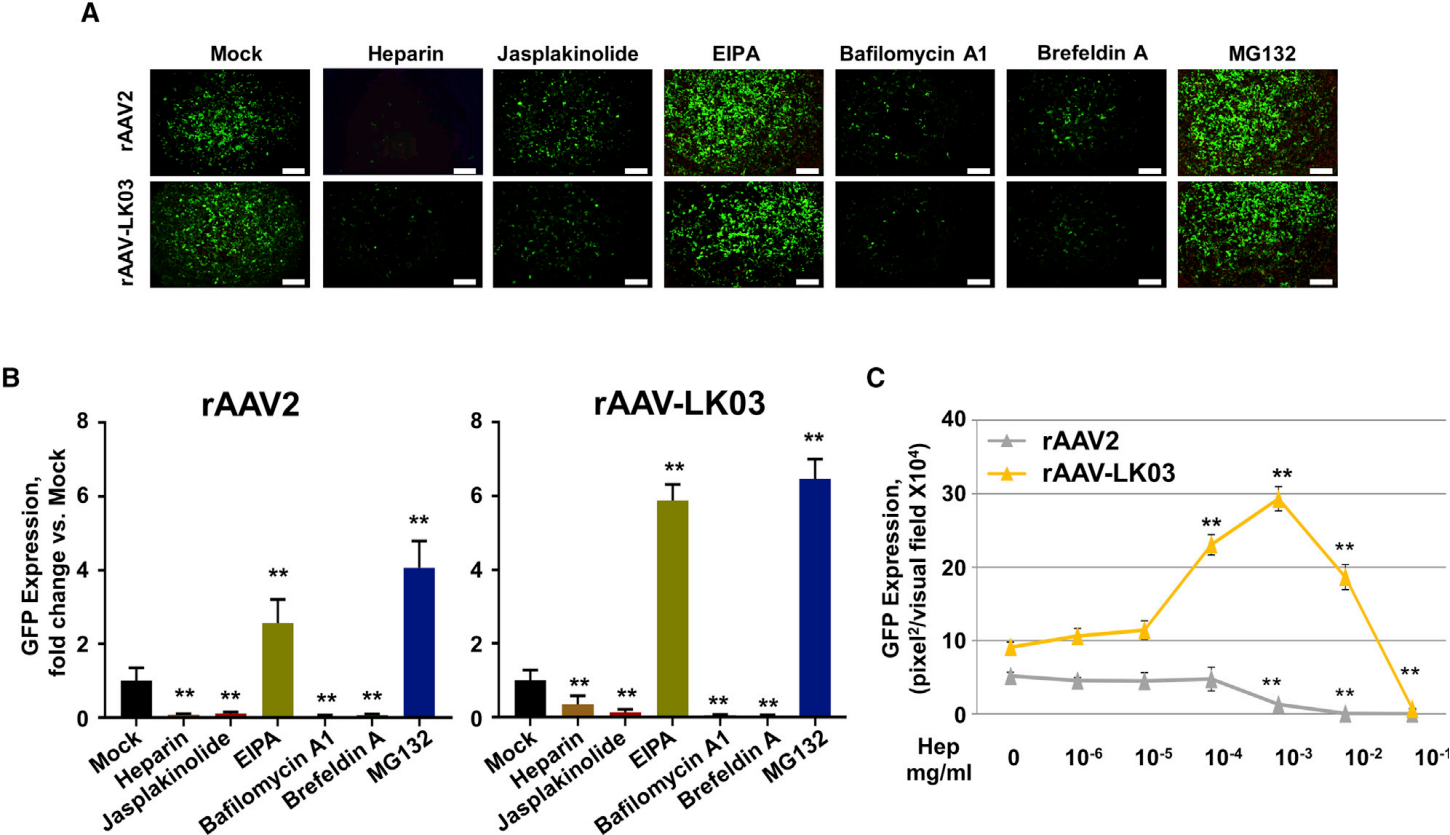
Transduction Efficiency of rAAV2-CMV-*egfp* and rAAV-LK03-CMV-*egfp* Vectors in Huh7 Cells, Treated by Various Specific Pharmacological Inhibitors (A) Huh7 cells were infected with GFP-expressing rAAV2 or rAAV-LK03 in the presence of specific inhibitors (100 μg/mL heparin, 2 μM jasplakinolide, 10 μM EIPA, 200 nM bafilomycin A1, 10 μg/mL brefeldin A, and 20 μM MG132). GFP expression was measured at 24 h post-infection by fluorescence microscopy. Scale bars represent 300 μm. (B) Quantitation of transduction efficiency of rAAV2 and rAAV-LK03 with different inhibitors. Data were normalized by rAAV2- and rAAV-LK03-infected and mock-treated groups, respectively. (C) Transduction efficiency of rAAV2 and rAAV-LK03 with different concentrations of heparin (0.1–100 μg/ml). All values shown are means ± standard deviations. ∗p < 0.05, ∗∗p < 0.01 versus mock.

The rAAV-LK03 vector was enriched from a DNA shuffling library for the purpose of deriving a clinical candidate vector for human liver-specific transduction. Human hepatocellular carcinoma (HCC) is a prevalent and highly malignant cancer throughout the world^17^^,^^18^ and is a target of rAAV-LK03. Thus, we used human Huh7 cells, a model HCC cell line, to investigate the effect of these above-mentioned inhibitors on rAAV-LK03 vector transduction. The transduction efficiency of both rAAV2 and rAAV-LK03 vectors was decreased by treatment with 100 μg/mL heparin (Figures 4A and 4B). Interestingly, low concentrations of heparin (0.1–10 μg/mL) boosted rAAV-LK03 transduction, while higher concentrations eventually inhibited it (Figure 4C). Meanwhile, rAAV2 transduction was inhibited by heparin in a dose-dependent manner, with more than 50% reduction at 1 μg/mL (Figure 4C). Similar to the above results using rAAV-DJ vectors in HEK293 cells, treatment with jasplakinolide, bafilomycin A1, and brefeldin A significantly decreased the transduction efficiency of rAAV-LK03 vectors. Thus, we concluded that endosome acidification and Golgi apparatus were involved in retrograde transport pathways of rAAV-LK03 vectors. MG132 improved the transduction efficiency of rAAV-LK03 vectors, indicating the role of escape from proteasomal degradation. It is also noteworthy that EIPA significantly increased rAAV-LK03 vector transduction in the Huh7 cells. This opposite effect (Figure 1A versus Figure 4A) indicated the possibility of the involvement of distinct host cell-specific entry pathways for the two vectors, as suggested by others.^19^

### Site-Directed Mutagenesis of rAAV-LK03 Capsid Improves Transgene Expression *In Vitro*

**Figure 5:**
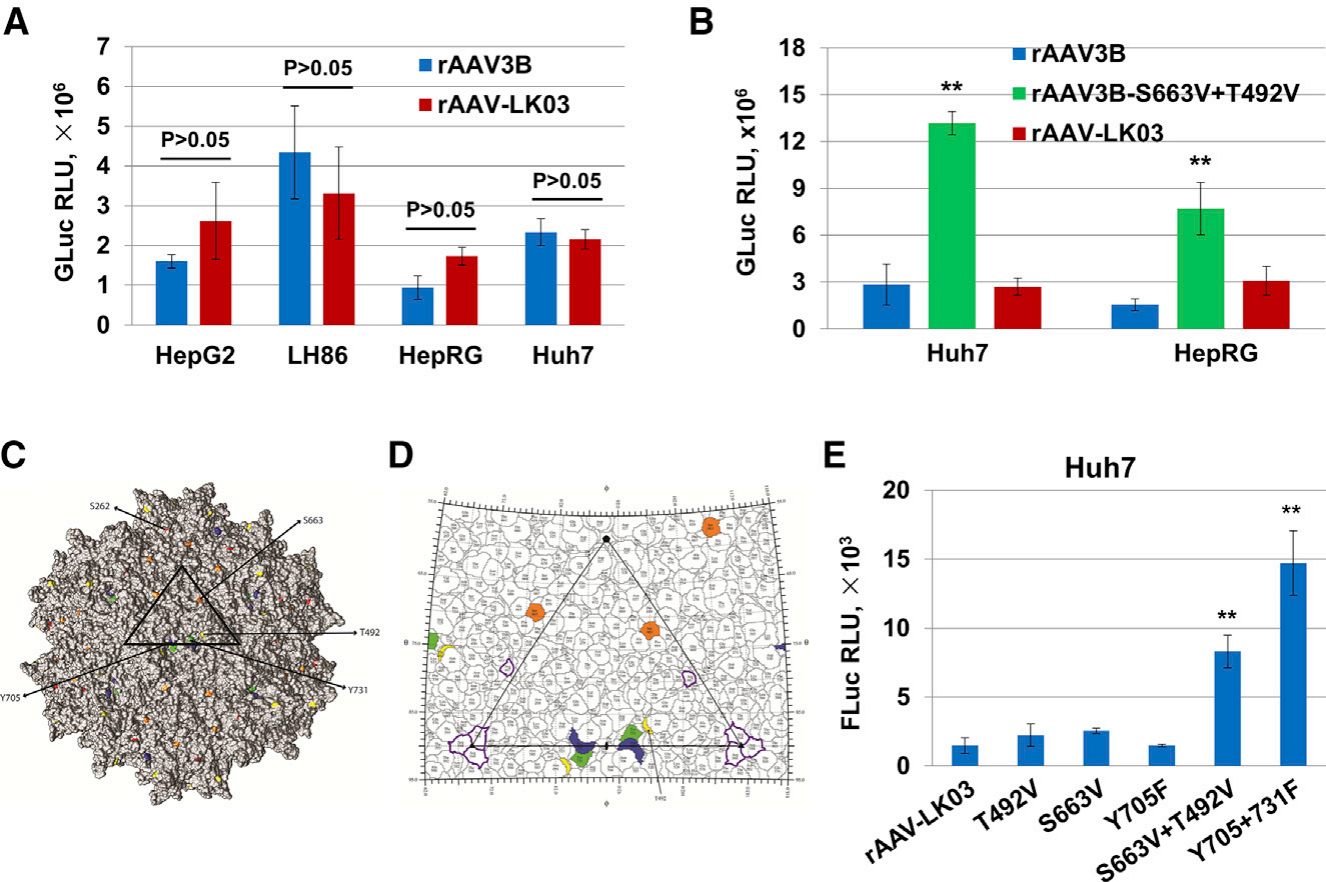
Transduction Efficiency of rAAV3B, rAAV-LK03, and Their Capsid-Modified Counterparts in Human Hepatic Cell Lines *In Vitro* (A and B) Transgene expression of (A) rAAV-LK03-CBA-*gluc*, rAAV3B-CBA-*gluc*, and (B) capsid-modified rAAV3B-S663V+T492V-CBA-*gluc* vectors in the indicated human hepatocellular carcinoma cell lines. The GLucs were measured in the supernatant of cell cultures that were transduced with the viral vectors at an MOI of 5 × 10^3^ vg/cell for 48 h. (C and D) Capsid structure of AAV3B (PDB: 3KIC) with the viral asymmetric unit outlined in black (bold triangle). Key residues for the improved transduction within rAAV3B and rAAV-LK03 are highlighted: T492 (yellow), S663 (orange), Y705 (blue), and Y731 (green). (C) Surface representation of the viral capsid viewed down the 2-fold (2f) axis of symmetry. From this orientation S262 is visible (red) tucked underneath the base of the 3-fold (3f) protrusions. S262 is important for species-limited transduction of human hepatocytes. (D) The spherical viral capsid projected onto a two-dimensional roadmap and viewed from the exterior capsid surface. Three out of four residues involved in proteasome trafficking cluster near the icosahedral 2f symmetry axis (T492, Y705, and Y731). However, S663 lies within the canyon that surrounds the 5-fold (5f axis). Residues implicated in receptor binding are located within 3f protrusions and are outlined in purple (R447 and R594). 2-fold, black-filled elliptoid; 3-fold, black-filled triangle; 5-fold, black-filled pentagon. (E) Transgene expression of capsid-modified rAAV-LK03-CBA-*fluc* vectors in Huh7 cells. The FLucs were measured in Huh7 cells that were transduced with the rAAV-LK03 mutants at an MOI of 5 × 10^3^ vg/cell for 48 h. All values shown are means ± standard deviations. ∗p < 0.05, ∗∗p < 0.01 versus rAAV-LK03.

In view of a similar response to treatment with heparin by both rAAV3B and rAAV-LK03,^20^ and since both capsids share 98.9% homology of the amino acid sequence,^11^ we hypothesized that these two vectors may use a similar infectious entry pathway in targeting cells. Indeed, in our initial comparison using four different human hepatic cell lines (HepG2, LH86, HepRG, and Huh7) *in vitro* (Figure 5A), no significant differences were observed between the rAAV3B and rAAV-LK03 vectors carrying a self-complementary *Gaussia* luciferase (*gluc*) gene under the control of a CMV enhancer/chicken β-actin hybrid promoter (CBAp). In addition, the optimized rAAV3B capsid mutant vector, rAAV3B-S663V+T492V, mediated significantly increased transgene expression versus both WT rAAV3B and rAAV-LK03 vectors (Figure 5B). Thus, we modified the rAAV-LK03 capsid proteins with specific surface-exposed tyrosine (Y), serine (S), and threonine (T) residues based on our previously published studies with rAAV3B vectors.^21^, ^22^, ^23^ In Figure 5C, the mutated sites (T492, S663, Y705, and Y731) are highlighted on the capsid surface of rAAV3B (RCSB PDB: 3KIC).^24^ Each amino acid is surface accessible and has been labeled within the viral asymmetric unit (outlined, bold triangle). These amino acids lie in close proximity to the 2-fold (2f) depression or surround the 5-fold (5f) channel (Figure 5D). The WT and mutant vectors were packaged with a single-stranded firefly luciferase (*fluc*) gene under the control of CBAp. Huh7 cells were used to evaluate the transduction efficiency of these mutant vectors. Consistent with our previously published studies with rAAV3B vectors,^22^ the rAAV-LK03-Y705+731F mutant vector was ∼10-fold more efficient in transducing Huh7 cells compared with the rAAV-LK03 vector. The rAAV-LK03-S663V+T492V mutant vector also increased the transduction efficiency by ∼6-fold compared with the rAAV-LK03 vector (Figure 5E), albeit the rAAV3B-S663V+T492V mutant vector was ∼15-fold more efficient than the rAAV3B vector.^22^

### *In Vivo* Characterization of the rAAV-LK03-Y705+731F Vectors

**Figure 6:**
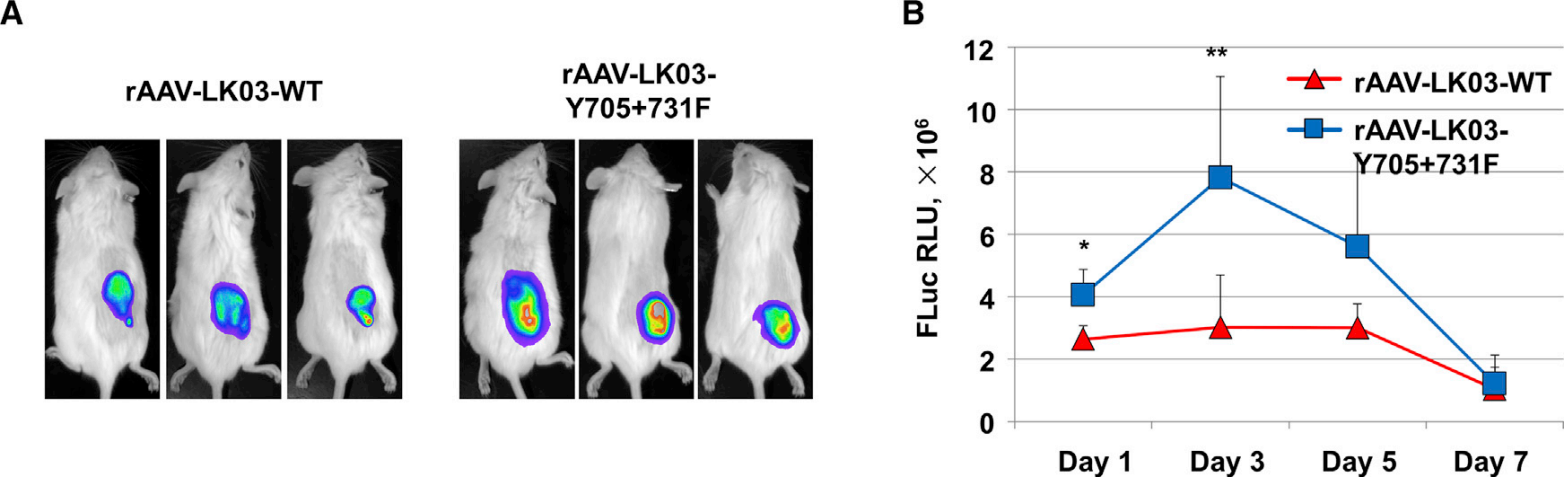
rAAV-LK03-Y705+731F Vectors Mediated Higher Transduction Efficiency in a Human HCC Xenograft Model following Intravenous Vector Injection Huh7 tumor-bearing NSG mice were tail vein injected with rAAV-LK03-CBA-*fluc* or of rAAV-LK03-Y705+731-CBA-*fluc* vectors at 1 × 10^11^ vg/mouse. (A) Whole-body bioluminescence images of NSG mice were obtained at 3 days post-injection. (B) Quantitative data of bioluminescent signals in mouse tumor xenografts at days 1, 3, 5, and 7 after vector administration. All values shown are means ± standard deviations. ∗p < 0.05, ∗∗p < 0.01 versus WT.

We also wanted to evaluate the transduction efficiency of the optimized rAAV-LK03-Y705+731F vectors in human HCC tumors in a murine xenograft model *in vivo*. To this end, we first determined that there was no difference in rAAV-LK03 vector-mediated transgene expression in the HCC tumor with or without Matrigel, which made tumor cells grow faster *in vivo* (data not shown). Subsequently, 1 × 10^11^ vg/mouse of single-stranded rAAV-LK03 or rAAV-LK03-Y705+731F vectors carrying the *fluc* gene under the control of CBAp were injected intravenously into human Huh7 tumor-bearing NSG mice. Whole-body bioluminescence imaging was performed 1, 3, 5, and 7 days after vector administration. We observed that the fluc transgene expression was significantly increased with the rAAV-LK03-Y705+731F mutant vector compared with the rAAV-LK03 vector (Figure 6A). The highest level of transgene expression with the mutant vector was on day 3 post-injection, which declined over time from both vectors due to vector genome dilution and the rapid proliferation rate of tumors (Figure 6B). It is noteworthy that there was no observable transgene expression in the normal liver of mice during 1-day and 7-day post-vector administration through the tail vein, an observation that is consistent with previously published studies.^11^^,^^22^ Taken together, these data further corroborate that rAAV3B and rAAV-LK03 vectors are functionally similar, and that specific modifications in the capsids of both vectors result in significantly increased transduction efficiency in human hepatic cells, both *in vitro* and *in vivo*.

## Discussion

High-resolution structural studies of AAV capsids have contributed enormously not only to our fundamental understanding of AAV biology, but also in the development of rationally designed rAAV vectors with enhanced transduction efficiency and neutralizing antibodies avoidance.^25^ To our knowledge, most, if not all, previous rational designs have been applied to the naturally occurring AAV serotype capsids. The present study was aimed to evaluate whether capsids derived and selected from AAV capsid libraries could be further improved. To this end, based on our long-term interest in the development of human liver-tropic AAV vectors,^21^, ^22^, ^23^^,^^26^^,^^27^ we chose to focus on two such library-derived hepatotropic vectors, rAAV-DJ^8^ and rAAV-LK03.^11^

We recently reported that rAAV-DJ is powerful to transduce mouse and human liver ductal organoids, and that site-directed mutagenesis of specific amino acids in the rAAV-DJ capsid further increased its transduction efficiency.^28^ These and other rAAV-DJ-based single point mutation studies^29^ prompted us to systemically introduce single, double, and triple mutants into the rAAV-DJ capsid. The mutant sites in our study were all reported to increase the transduction efficiency of rAAV2 vectors. For instance, in an early report in 2006, Lochrie et al.^30^ compared S267T and S267A on the AAV2 capsid and concluded that the rAAV2-S267T mutant exhibited the largest increase in transduction. Although the underlying mechanism remained unclear, most of these mutations also augmented rAAV-DJ vector-mediated transgene expression in HEK293 cells. However, some of the capsid mutants failed to transduce human liver cancer cell lines. As stated above, rAAV vector transduction is highly dependent on host cell type and the intracellular environment.^19^ Thus, additional studies are warranted to gain a better understanding of the molecular mechanisms underlying rAAV-DJ vector binding, entry, trafficking, phosphorylation, ubiquitination, and proteasome-mediated vector degradation in various cells and tissues.

rAAV-LK03 was derived from selection in “humanized” mouse liver.^11^ It is selectively tropic for human hepatocytes, a property it shares with rAAV3B.^23^ We also observed that rAAV-LK03 possesses a phenomenon that was thought to be unique to the rAAV3B vector, in that the vectors based on this serotype mediate enhanced and reduced transduction in the presence of low and high heparin, respectively.^20^ It has been reported that rAAV-LK03 vectors transduce human hepatocytes 5.6-fold more efficiently than do rAAV3B vectors *in vitro*, and ∼12-fold more efficiently in humanized mice *in vivo*.^11^ However, we were not able to document any significant differences in the transduction efficiencies of rAAV3B and rAAV-LK03 vectors in four different human liver cancer cell lines (Huh7, HepG2, LH86, and HepRG) *in vitro* (Figure 4A). This was further corroborated by the fact that when specific single (Y705F, S663V, and T492V) and double (Y705+731F and S663V+T492V) mutations were introduced into the LK-03 capsid, at least two double mutations (Y705+731F and S663V+T492V) significantly increased the transduction efficiency of LK-03 vectors by ∼10-fold and ∼6-fold, respectively (Figure 4E).

Several of the residue differences between rAAV3B and rAAV-LK03 (K26, V29, Q31, R41, E67, Q105, and I125) exist within the unique region of VP1, known as VP1u.^11^ These amino acid positions are not ordered in the available crystal structure of AAV3B.^24^ The remaining single amino acid difference, N735, is buried underneath the 3-fold (3f) protrusions on the capsid interior in a distinct location (≥12 Å) from any important residues identified in our study. However, a structural analysis of the these two rAAV3B capsid structure reveals that Y705 and Y731 lie adjacent to the 2f axis of symmetry within close proximity to each other with Y731 localized four residues away. Interestingly, there are several tyrosine and phenylalanine residues clustered at the 2f here on the capsid surface (365, 613, 701, 705, 713, 721, and 731) that are located within 4.27–9.85 Å of each other. This arrangement of aromatic amino acids within a cavity of the 2f depression forms a hydrophobic patch on either side of the icosahedral 2f axis (Figure 4D). Therefore, mutagenesis of Y705F, Y731F, and additionally T492V (positioned just above this cavity) increases the number of hydrophobic interactions occurring in this region of the capsid. Alternatively, S663 does not appear to interact directly with these residues, although its location at the base of the 5f channel suggests a role in extrusion of VP1u and potential unseen interactions with remaining N-terminal residue differences between rAAV3B and rAAV-LK03. Furthermore, VP3 residues controlling proteasome trafficking in the current study function independently of amino acids previously implicated in either AAV3B HS receptor binding (R4497 and R594) or selective tropism for human hepatocytes (S262) as visualized in Figure 4D.^11^^,^^24^

Taken together, our studies not only demonstrate that site-directed mutagenesis of specific surface-exposed amino acid residues on non-natural AAV capsids retain their remarkable tropism, but they also suggest that this strategy should be applicable to other non-natural AAV variants for their optimal use in human gene therapy.

## Materials and Methods

### Cell Cultures

The human embryonic kidney cell line HEK293 and human hepatocellular carcinoma cell line HepG2 were purchased from the American Type Culture Collection (ATCC, Manassas, VA, USA). The human hepatocellular carcinoma cell line Huh7 was obtained from Dr. Chen Liu’s laboratory at the Cancer Institute of New Jersey, Rutgers Health, and was previously described.^31^ All cells were maintained as monolayer cultures in Dulbecco’s modified Eagle’s medium (DMEM), supplemented with 10% fetal bovine serum (FBS), 100 U/mL penicillin, and 100 mg/mL streptomycin (Multicell Wisent, Saint-Jean-Baptiste de Rouville, QC, Canada). All cells were cultured in a humidified atmosphere at 37°C in 5% CO_2_.

### Construction of AAV-DJ and LK03 Mutants

Mutations in rAAV-DJ and rAAV-LK03 Rep/Cap plasmids were performed by a site-directed mutagenesis kit (BBI Life Science, Shanghai, China), according to the manufacturer’s instructions. The primers used for site-directed mutagenesis of rAAV-DJ and rAAV-LK03 capsids are shown in Table 2. Briefly, the two complementary mutagenic primers with centrally located mutation sites were used to amplify the entire target vectors through using the Pfu DNA polymerase. The PCR extension reactions generated large numbers of mutated plasmids with staggered nicks. Then DpnI restriction endonuclease was used to digest the residual methylated template and any hemimethylated DNA. The nicked vector DNA was transformed into competent cell DH5α, which can repair the nicked vector. The desired point mutation was corroborated by DNA sequencing.

**Table 2:** Primer Sequences for Site-Directed Mutagenesis of rAAV-DJ and rAAV-LK03 Capsid

Mutants	Forward Primer Sequences (5′→3′)	Amino Acid Change: WT → Mutant
rAAV-DJ		
S269T	AGCACATCTGGAGGATCTACAAATGACAACGCCTACTTCG	TCA (S) → ACA (T)
Y446F	TGATTGACCAGTACCTGTACTTCTTGTCTCGGACTCAAAC	TAC (Y) → TTC (F)
T493V	GCAGCGAGTATCAAAGGTCTCTGCGGATAACAACAACAGT	ACA (T) → GTC (V)
D496E	CGAGTATCAAAGACATCTGCGGAGAACAACAACAGTGA	GAT (D) → GAG (E)
W504A	ACAACAACAGTGAATACTCGGCAACTGGAGCTACCAAGTA	TGG (W) → GCA (A)
Y706F	GATCCAGTACACCTCCAACTTCTACAAATCTACAAGTGTG	TAC (Y) → TTC (F)
Y732F	TGAACCCCGCCCCATTGGCACCCGTTTCCTCACCCGTAAT	TAC (Y) → TTC (F)
rAAV-LK03		
T492V	GCAACAGAGACTTTC AAAGGTTGCTAACGACAACAACAA	ACT (T) → GTT (V)
S663V	AA ATCCTCCGACGACTT TCGTCCCGGCCAAGTTTGCTTC	AGC (S) → GTC (V)
Y705F	GATTCAGTACACTTCCAACTTCAACAAGTCTGTTAATGTGG	TAC (Y) → TTC (F)
Y731F	TCGCCCCATTGGCACCCGTTTCCTTACCCGTCCCCTGTAA	TAC (Y) → TTC (F)

### Recombinant AAV Vector Production

Recombinant AAV vectors were generated as previously described.^32^ Briefly, HEK293 cells were transfected with three plasmids using polyethyleneimine (PEI; linear, molecular weight [MW], 25,000; Polysciences). Cells were harvested at 72 h post-transfection, followed by three rounds of freeze-thawing and treated with Benzonase (50 U/mL crude lysate) for 1 h at 37°C. The crude viral vector stocks were purified by iodixanol (Sigma) gradient centrifugation and ion exchange column chromatography (5-mL HiTrap SP HP, GE Healthcare). Then, the virus was concentrated, and the buffer was exchanged in three cycles to lactate Ringer’s solution using centrifugal spin concentrators (Apollo, 150-kDa cutoff, 20-mL capacity, Cambio Ltd, Dry Drayton, Cambridge, U.K.). The physical particle titers of highly purified rAAV vector stocks were determined by qPCR.

### rAAV Vector Transduction Assays *In Vitro*

Cells were seeded in 96-well plates at a final density of 1 × 10^4^ cells/well in complete medium and were transduced with AAV vectors at an MOI of 5,000 vg/cell in DMEM without FBS and antibiotics for 2 h. After transduction, the cells were washed by PBS twice and incubated for an additional 72 h in complete DMEM. Then, the expression of the reporter genes was analyzed by direct fluorescence imaging, flow cytometry, or an injector-equipped luminometer.

### Pharmacological Inhibitors Assays

Cells were seeded in 96-well plates at a concentration of 1 × 10^4^ cells/well in complete DMEM. Twenty-four hours later, cells were switched to premixture of vectors and heparin sodium salt for 2 h and then incubated for an additional 24 h in complete medium for analysis of EGFP levels by fluorescence imaging. Additionally, other inhibitors were used to treat cells for 1 h prior to infection, and the expression of EGFP at 24 h post-infection was analyzed by direct fluorescence imaging.

### rAAV Binding and Internalization Assays

To assess binding, cells were incubated for 30 min at 4°C. Then, cells were infected with AAV at an MOI of 2,500 vg/cell of AAV at 4°C for 1 h and were gently rinsed three times with ice-cold PBS. Total cell DNA was extracted using a genomic DNA mini preparation kit with a spin column kit (Beyotime, Shanghai, China) following the manufacturer’s instructions. Cell-associated AAV DNA was quantified by a qPCR assay as previously described, using a 2 × T5 Fast qPCR mix (SYBR Green I) kit (Tsingke Biological Technology, Shanghai, China) with transgene-specific primers.

To assess internalization, cells were incubated at 37°C for 1 h in 5% CO_2_ after infection at 4°C for 1 h, treated with trypsin to remove surface-bound virions that did not internalize, and washed three times with ice-cold PBS. Isolated total DNA was quantified by qPCR with transgene-specific primers.

### Animals and rAAV Vector Administration

All animal experiments were approved by an Institutional Animal Care and Use Committee. All procedures were performed according to the guidelines for animal care specified by the Animal Care Services at the University of Florida (Gainesville, FL, USA) or Fudan University (Shanghai, China). Every effort was made to minimize pain and suffering. Six- to 8-week-old nonobese diabetic/severe combined immunodeficient, interleukin-2γ-deficient (NSG) mice were purchased from Jackson Laboratory and maintained by the Animal Care Services at the University of Florida College of Medicine (Gainesville, FL, USA). Six- to 8-week-old C57BL/6J mice were purchased from Beijing Vital River Laboratory Animal Technology (Beijing, China). rAAV vectors were diluted in 200 μL of PBS and injected intravenously via the tail vein at a dose of 10^11^ vg/mouse.

### *In Vivo* Liver Sections

Liver tissues were isolated from the mice and fixed in 4% paraformaldehyde overnight. After 30% sucrose dehydration, tissues were embedded with OCT compound at a low temperature and sliced into 5-μm-thick sections. The expression of GFP in liver was analyzed by an Olympus IX73 microscope (×10 objectives). The frozen liver sections were fixed and permeabilized in cold acetone and blocked with 10% goat serum diluted with PBS. To detect the bile duct-specific cytokeratin 19 (CK19), the antibody (1:500 dilution, ab52625, Abcam, USA) and the goat anti-rabbit immunoglobulin G (IgG) (H+L) cross-adsorbed secondary antibody (1:10,000 dilution; A-21428, Invitrogen, USA) were used. For detection of the nuclear location, DAPI (D9542, Sigma-Aldrich, USA) was used. After staining, an Olympus IX73 microscope was used to observe the fluorescence of liver, and the images were taken by ×40 objectives.

### Viral Vector *In Vivo* Biodistribution Assays

DNA was extracted from isolated tissues by a DNA extraction kit (TSP201-50, Tsingke, China). Absolute quantification of vector genomes per 100 ng of DNA was determined by qPCR against CMVp content using a SYBR Green kit (TSE201, Tsingke, China). To make the standard curve, plasmid pdsAAV-CMV-*egfp* was used as standard sample (1, 0.1, and 0.01 ng/well), and 200 ng of liver DNA isolated from PBS-injected mice was added into the standard sample as the negative control. Primers sequences are as follows: forward, 5′-CATTGACGTCAATAATGACG-3′; reverse, 5′-TGTACTGGGCATAATGCCAG-3′.

### *In Vivo* Tumor Xenografts

Ten million Huh7 cells were collected and resuspended in 200 μL of PBS containing 50% Matrigel (354248, Corning, Bedford, MA, USA), followed by subcutaneous injection at the right flank of the mice. Ten days after inoculation while tumor volumes (*V* = a × *b*^2^/2, where *a* and *b* represent length and width, respectively) were close to 100 mm^3^, the mice were randomly divided into treatment groups (n = 3 per group).

### *In Vivo* Firefly Luciferase Imaging

*In vivo* bioluminescence images were taken as previously reported.^33^ Briefly, images were immediately acquired during a period of 5 min using a Xenogen IVIS Lumina system (Caliper Life Sciences). Signal intensity was quantified using the camera control program, Living Image software, and presented as photons/s/cm^2^/steradian.

### Statistical Analysis

All data are presented as the mean ± standard deviation. The different groups’ comparisons were performed by using a Student’s t test or analysis of variance (ANOVA). p < 0.05 was considered statistically significant.

## Author Contributions

G.R., X.C., and Y.X. performed *in vitro* and *in vivo* experiments and were involved in data analysis. Q.Z. and J.X. produced viral vectors and were responsible for the vector quality control. C.Y. performed binding and internalization assays. F-X.Y. and S.Q. performed immunofluorescence staining of liver sections. N.P. and M.A.-M. provided the capsid structure and two-dimensional surface roadmap. M.A.-M, A.S., and C.L. designed experiments, analyzed data, and wrote the manuscript.

## Conflicts of Interest

A.S. is a cofounder of, and holds equity in, Lacerta Therapeutics, aaVective, KASHX Bio, and Nirvana Therapeutics, and is an inventor on several issued patents on recombinant AAV vectors that have been licensed to various gene therapy companies. M.A.-M. is a SAB member for Voyager Therapeutics, Inc., and AGTC, has a sponsored research agreement with AGTC and Voyager Therapeutics, and is a consultant for Intima Biosciences, Inc. M.A.-M. is a co-founder of StrideBio. C.L., A.S., and M.A.-M. have Intellectual Property (IP) on AAV vectors. The remaining authors declare no competing interests.
